# Bridging Targeted and Untargeted Mass Spectrometry-Based Metabolomics via Hybrid Approaches

**DOI:** 10.3390/metabo10090348

**Published:** 2020-08-27

**Authors:** Li Chen, Fanyi Zhong, Jiangjiang Zhu

**Affiliations:** 1 Department of Human Sciences, The Ohio State University, Columbus, OH 43210, USA; chen.10289@osu.edu; 2 James Comprehensive Cancer Center, The Ohio State University, Columbus, OH 43210, USA; 3 Department of Chemistry and Biochemistry, Miami University, Oxford, OH 45056, USA; zhongf@miamioh.edu

**Keywords:** metabolomics, hybrid approaches, broad metabolite coverage, quantitative analysis, dynamic range, repeatability, identification

## Abstract

This mini-review aims to discuss the development and applications of mass spectrometry (MS)-based hybrid approaches in metabolomics. Several recently developed hybrid approaches are introduced. Then, the overall workflow, frequently used instruments, data handling strategies, and applications are compared and their pros and cons are summarized. Overall, the improved repeatability and quantitative capability in large-scale MS-based metabolomics studies are demonstrated, in comparison to either targeted or untargeted metabolomics approaches alone. In summary, we expect this review to serve as a first attempt to highlight the development and applications of emerging hybrid approaches in metabolomics, and we believe that hybrid metabolomics approaches could have great potential in many future studies.

## 1. Introduction

Metabolomics, an “-omic” science in systems biology, is widely used to assess and evaluate both the endogenous and exogenous metabolites present in a biological system [1]. Followed by its inception, scientists have been engaged in the development of metabolomics methods for the analysis of small molecular weight compounds in biological systems including biofluids, cells, tissues, and/or organisms [2,3]. Working with the sophisticated biological processes and systems, metabolomics can be a useful tool for biomarker discovery [4], disease diagnosis [5], and biochemical pathway elucidation [6]. In particular, the differentiation of metabolic responses between unperturbed and perturbed groups, such as between healthy control and patients with a particular disease, is frequently studied by metabolomics [7]. The complementary analytical techniques of nuclear magnetic resonance (NMR) and mass spectrometry (MS) are the most popular choices [8]. In general, NMR could measure metabolites up to the micromolar (μM) range or a few nmol at high fields using new cryoprobes [9,10], whereas MS permits the detection of down to pmol or nmol concentrations [11]. Meanwhile, due to the spurs of hyphenated MS instrument development, chromatography separation coupled with MS-based technologies has become the mainstream choice in metabolomics studies in recent years [12].

Generally, metabolomics studies can be accomplished using either a targeted approach or an untargeted approach. In most circumstances, targeted analyses focus on identifying and quantifying a limited number of metabolites [13]. For instance, a targeted analysis of 36 major metabolites from only 10 μL of whole blood can be achieved with good repeatability and stability [14]. A total of 245 standard compounds were used in a large-scale analysis of targeted metabolomics data from heterogeneous biological samples [15]. A targeted high-performance liquid chromatography (HPLC)-MS approach was used to identify 159 [16] reliable metabolites in serum; meanwhile, a gas chromatographymass spectrometry (GC-MS method was used to identify the changes in 58 metabolites in the wheat metabolome [17]. Further, some targeted metabolomics kits can quantity up to 500 compounds [18,19]. However, it is difficult to obtain all the required chemical standards for the metabolites of interest, therefore the coverage of detected metabolites in targeted metabolomics is generally limited. Different from a targeted approach, the untargeted metabolomics approach focuses on the simultaneous detection of many unknowns, which can provide a wide range of detection of metabolites/metabolic features with diverse chemical and physical properties. For example, an untargeted approach reported the annotation of more than 350 phenolics using ultra-high performance liquid chromatography (UHPLC) coupled with an electrospray ionization quadrupole time-of-flight MS (ESI-Q-TOF MS) [20]. In another orbitrap-based untargeted metabolomics analysis, a simulated database with a total of ~80,000 molecules of lipids was reported [21]. Despite these advantages in untargeted metabolomics, the compound identifications and quantifications remain challenging for all detected metabolites/metabolic features [22,23].

While both targeted and untargeted metabolomics have their strengths and weaknesses, the greatest challenge is maximizing the detection and accurate identification of thousands of metabolites and maintaining a decent detection dynamic range and quantification capability. The recent trend in metabolomics research indicated the need for bridging the two mainstream approaches to form a possibly revolutionary approach—a hybrid approach. In this mini-review, the advantages of bridging these two approaches are discussed in detail, and the analytical performances are compared. The workflow of novel metabolomics approaches, instruments utilized, data analysis strategies, and broad applications are reviewed. To this end, MS-based hybrid approaches in metabolomics papers published in the years 2012−2020 are discussed.

## 2. Hybrid Approaches and the Novel Workflow in Metabolomics

**Figure 1:**
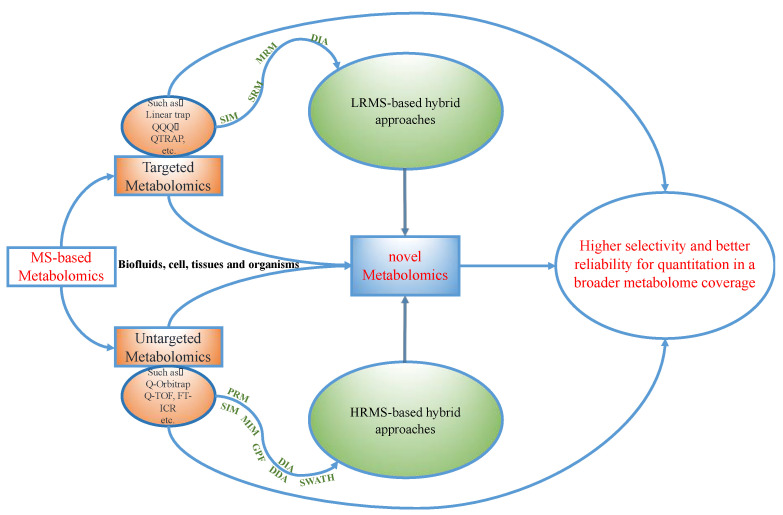
The workflow demonstrates the development process from targeted metabolomics and untargeted metabolomics to novel metabolomics analysis. Note: HRMS is commonly used in untargeted metabolomics; however, it could also be used in targeted metabolomics.

The emerging requests for handling complex metabolomics analysis highlighted the need to expand the metabolite coverage and quantitative assays among many other analytical considerations. Therefore, several hybrid approaches that bridge the targeted and untargeted approaches recently emerged and were broadly defined as the hybrid approaches in metabolomics (Figure 1). In general, the hybrid methods acquire ions lists or ion pairs lists from real samples (mostly pooled samples from all biological replicates) or databases and aim to qualify and quantify as many metabolites as possible. They usually consist of three major steps: (1) performing untargeted profiling or database searches to provide rich information on all possible ions based on high-resolution mass spectrometers (HRMS) and/or low-resolution mass spectrometry (LRMS); (2) conducting an ion selection process to generate the MS peak list, which generally includes the details of the precursor ion, product ion, and retention time; (3) the MS peak information generated in step 2 is imported into the instrument method of dynamic multiple reaction monitoring (MRM) or selected reaction monitoring (SRM) mode, selected ion monitoring (SIM) mode, etc. To avoid co-elution occurrence or the detection of low-abundance metabolites, time staggered or mass staggered ions lists are always built. Then, a sample might run several times follow the staggered lists. Subsequently, these new instrument methods are used for the hybrid analysis of complex metabolite matrices from different types of biological specimens [24,25].

**Figure 2:**
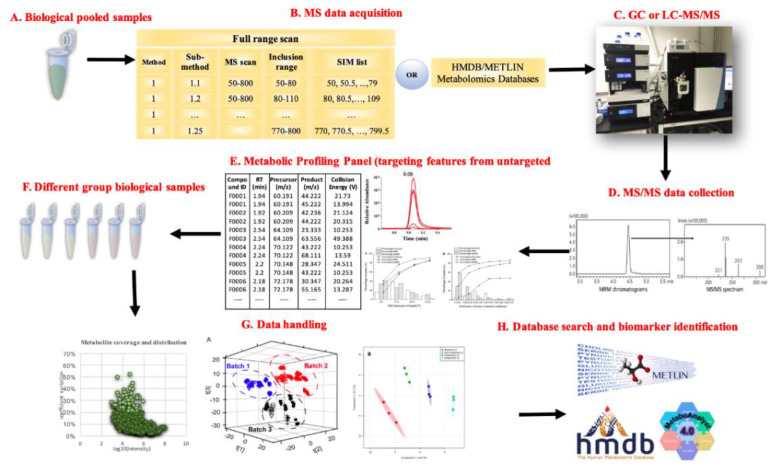
The workflow of novel metabolomics analysis with hybrid approaches [26,27,28,29].

While different types of hybrid approaches have been developed from a diverse set of metabolomics studies, these methods shared general developmental concepts. As demonstrated in Figure 2, the workflow generally started from sample preparation using pooled biological specimens (plasma, urine, tissues, etc.), then went through initial data acquisition, data driven-method development, data collection and processing, and metabolite characterization and identification/quantification. Below, we discuss the novel procedures of these hybrid approaches and compare them with the targeted/untargeted metabolomics workflow.

### 2.1. The Collection of Ion Pairs List in Hybrid Approaches

Although it is challenging to develop a method for the identification of all metabolites, a novel metabolomics method can provide global metabolome [30] information with the use of pooled samples from biological replicates [24], and collect a large amount of compound fragmentation information that could also be used for future structure elucidation and potential metabolite quantification. The key to this type of method is to get the characteristic ion-pairs from tens of thousands of MS^2^ spectra scanned from a real biological matrix (e.g., plasma, serum, and urine) [31]. Typically, ion pairs are acquired from metabolite standards in targeted metabolomics [32,33]; however, it is impossible to acquire standards for most of the metabolites encountered in metabolomics analyses. Although the surrogate standards [34] or a single standard per metabolite class [35] are commonly used in both targeted and untargeted metabolomics, the analysis is still restricted by the number of metabolites that can be detected in one large panel. Because the initial ion lists of hybrid approaches are established based on a full scan of the pooled biological samples or the reported database, this eliminated the reliance on chemical standards, which may not always be available. Meanwhile, it showed a higher selectivity and better reliability for quantitation in a broader metabolome coverage [27]. Researchers could use a variety of pooled biological samples for their hybrid method developments, such as rice seed [28], tobacco leaf [25,36], human serum/plasma [24,28,31,37,38,39], human urine [39,40,41], mouse serum/plasma [27,42,43], mouse brain and liver [44], cancer cell [44], and bacterial culture [29,45]. With the MS peak lists and ion pairs generated from the highly diverse set of small molecules within the studied biological matrix or reported database, hybrid methods in metabolomics permit the analysis of all metabolites in a given biological sample.

### 2.2. The Choices of Metabolomics Platforms in Hybrid Analyses

When choosing an MS instrument for developing hybrid approaches, HRMS, such as quadrupole time-of-flight (Q-TOF) mass spectrometry [46] and quadrupole-orbitrap (Q-Orbitrap) mass spectrometry [47], are often selected. These instruments directly challenge the LRMS, such as triple quadrupole mass spectrometry [24] and quadrupole trap (QTrap) mass spectrometry [48]. Hyphenated MS-based approaches are often favored. For example, GC-MS can be used but is generally limited to identify volatile and semi-volatile compounds for metabolic investigation, and extensive sample preparation, such as derivatization, is often required. Meanwhile, UHPLC/HPLC-MS gained popularity in hybrid approaches because of its quicker and less extensive extraction procedures and ability to identify and measure a broader range of compounds [49,50].

Generally, a single method cannot provide optimal detection for all metabolites in complex biological samples. Rather, samples are typically analyzed multiple times by one MS system or multiple MS systems in hybrid approaches. As shown in Figure 1 and Table 1, LRMS, HRMS, or a combination of both types of instruments can be used to implement metabolomics research. The following discussion will introduce the development of hybrid approaches, including globally optimized targeted (GOT) methods [24,29,45,51], pseudo-targeted methods [25,27,28,37,40,44], gas-phase fractionation (GPF) methods [38], data-independent targeted quantitative metabolomics (DITQM) [39], information-dependent acquisition (IDA) [31], and simultaneous targeted quantification and untargeted metabolomics [26], in metabolomics.

#### 2.2.1. The Single Usage of LRMS with Hybrid Approaches

The GOT- QQQ MS was exemplified by the recent development and application studies from several research groups [24,45,51]. During the GOT-QQQ MS development, the ion pairs were globally searched from real samples or reported databases, such as the Human Metabolome Database (HMDB) and METLIN. Then, the scheduled MRM/SRM mode was used to maximize the number of ion pairs detected in each measurement. This approach was developed to acquire MRMs/SRMs without a lot of chemical standards and independent of strict mass resolution/accuracy. It was well qualified to detect not only well-known metabolites but also unknowns or less-studied small molecules with better sensitivity and repeatability. However, during the process, the scheduled MRM/SRM methods were generally divided into multiple sub-methods; these methods need to be performed with repeated sample injections and prolonged experimental time. Meanwhile, additional experimental time was required to optimize the many MRMs/SRMs for particular sample types. Notably, a metabolite database is not an established method for scoring the probability of identification. When ion pairs are searched from the database, the risk of including false positives is high.

The combination of global profiling and targeted approaches in GOT-MS enabled the detection of the detailed structure of a large number of unknowns, and excellent quantification and identification potential in this type of hybrid study. The main procedures were: (1) a SIM was performed, and relatively high intensity and good signal-to-noise ratio were used as cutoffs to select precursor ions; (2) MS/MS was used to scan the product ions, and different collision energies were used for optimization purposes; (3) the selected ion pairs from previous steps were summarized in one instrument method, and the scheduled MRM mode was then used to maximize the number of MRMs in each measurement. After running the same samples, a GOT-MS analysis resulted in 26 MRMs/metabolites with fold changes >2 and *P* < 0.05, compared to only one significant metabolite detected for the Q-TOF and one for the traditional large targeted assay [24].

Subsequently, the GOT-MS was optimized to the database-assisted dGOT-MS [51]. The databases contain comprehensive mass spectra from a large number of small-molecule metabolite standards in both positive and negative ionization modes with multiple collision energies [56,57], and they were used in the dGOT-MS development. It was reported that dGOT-MS could cover an extensive range of metabolites (including lipids) from various types of biological samples. The overall analytical strategy of dGOT-MS is as follows: (1) precursor ions and product ions were acquired from databases; (2) the optimization of the retention and MRM/SRM parameters was conducted; (3) a four-level system for metabolite identification was decided and listed; (4) validated dGOT-MS was used to discover potential biomarkers in biological samples. In the dGOT-MS study, the traditional large-scale targeted detection, limited by the availability of standards for the predefined metabolites, resulted in the discovery of only five potential biomarkers with *p* < 0.05 and FC > 1.5. In contrast, 28 potential biomarkers (*p* < 0.05, FC > 1.5) were discovered using the novel dGOT-MS approach. These findings suggest that dGOT-MS is a highly useful approach for biomarker discovery related to breast cancer.

In addition to dGOT-MS, the GOT-MS was also modified by time staggered/mass staggered (ts/ms)GOT-MS [45]. The scheduled SRM or MRM was optimized by tsGOT and msGOT. For the tsGOT, all the SRM transitions were sorted from the smallest retention time to the largest retention time, and for the msGOT all the SRM transitions were sorted from the smallest precursors to the largest. In the ts/ms-GOT-MS analyses, 22.45%, 39.74% 54.90%, and 51.97% of peaks from the analyzed bacterial samples were detected with a CV < 10% by the targeted analysis, GOT-MS, tsGOT-MS, and msGOT-MS methods, respectively. The number of detectable bacterial metabolites from at least 75% of the samples was reported as 72, 273, 296, and 355 from the targeted analysis, GOT-MS, tsGOT-MS, and msGOT-MS, respectively. When the biomarker selection criteria of a 1.3-fold change and *t*-test *p*-value < 0.05 were applied, the number of metabolite biomarkers detected by the targeted metabolic profiling, GOT-MS, tsGOT-MS, msGOT-MS was 50, 198, 214, and 260, respectively [45].

#### 2.2.2. The Single Usage of HRMS with Hybrid Approaches

HRMS full scan methods are commonly used in untargeted metabolomics analysis [58]. However, there are single HRMS-based hybrid metabolomics approaches reported in the past few years. For instance, a hybrid approach to transforming an untargeted metabolic profiling method using the retention time locking (RTL) of SIM mode was reported [25]. In this study, the MS data were acquired with GC-TOF-MS in the full scan mode, and an algorithm based on the automated mass spectral deconvolution and identification system (AMDIS) was used to keep the most abundant peak and decrease the error or duplication of the deconvolution and detection results. However, the ion selection strategy was complex, and some false characteristic ions could still arise from the imperfect algorithm. Then, all the ions detected in the SIM mode were divided into several groups based on their retention time. Overall, the time-divided SIM method showed better sensitivity than those of the full scan-based total ion current (TIC) and extracted ions current (EIC) methods, and 167, 151, and 138 of the significantly different components (*p* < 0.05) in tobacco leaf were screened out using the data of the SIM, EIC, and TIC, respectively. Meanwhile, more than 36% (compared with 16% for both the EIC and TIC methods) of the components had a relative standard deviation (RSD) of less than 5%, and more than 93% of components (compared with 88% for the EIC method and 81% for the TIC method) had an RSD of less than 20% [25].

GPF-enhanced metabolite detection and identification using an LC-Q-TOF-MS instrument was another example of hybrid methods that used a single HRMS platform [38]. GPF was a procedure that could effectively utilize different MS/MS parameters across an analytical batch, or perform repetitive injections of the same sample using exclusion or inclusion criteria to select precursor ions. Specifically, the methods that were based on the different inclusion criteria for the selection of precursor ions to undergo MS/MS fragmentation were combined and programmed. The GPF protocols expanded the metabolite coverage (particularly with coeluting compounds) and provided MS/MS information for at least 80% of all detected entities, while in contrast the conventional auto-MS/MS mode generally could only provide 48–57%.

The other hybrid approach using the HRMS platform was the UHPLC-Q-TOF-MS-based pseudotargeted method with time-staggered ion lists [27]. The key step in this approach was to establish a target ion list in the multiple ion monitoring (MIM) mode, in which the precursor to precursor ion transitions were monitored using the “TOF MRM” functionality. The MS data were acquired from *m*/*z* 100 to *m*/*z* 1000 using the full scan mode, then the detected peaks were extracted and aligned. Next, based on the staggered time points, the original ion list was divided into three separate ion lists and imported into the method editor for batch analysis of the biological samples. The triggered time for each target ion was defined as retention time (RT) ± 0.15 min, and its collision energy was set to the low energy of 3 V to obtain the highest response. The approach exhibited better repeatability and a wider linear range than the traditional untargeted metabolomics; besides, a broader metabolite coverage (compared to single MIM mode) was observed. However, the established ts-MIM-based pseudotargeted metabolomics method has a major limitation, in that the loss of MS/MS information could happen due to the low collision energy used. In comparison, a target-direct data-dependent acquisition (tDDA) with time-staggered precursor ion lists (tsDDA) method can be used to improve the performance of the MS/MS acquisition. After untargeted analysis with a full scan, the MS data can be filtered to get a tDDA inclusion list. Then, the tDDA was split into three time-staggered ion lists to get tsDDA. Unlike the conventional DDA that automatically fragments the most abundant ions, tDDA, or tsDDA highlighted the features of interest regardless of their peak abundance and reduced the initiation of unnecessary MS/MS events. In these studies, plasma samples were used to evaluate and compare the performance of the MS/MS acquisition using the conventional DDA, tDDA, and tsDDA, and 97.4% and 95.4% of the selected ions in the positive and negative ion mode of tsDDA analysis have an RSD value of less than 20%, respectively [42]. Compared to the conventional DDA, the ts-DDA also demonstrated superior performance in the high co-elution zones of plasma samples (especially for the metabolites of low abundance). Furthermore, the integration of tsMIM and tsDDA into one workflow could improve the data acquisition in UHPLC-Q-TOF-MS-based hybrid analyses [43]. Nevertheless, even under optimized parameters, a reduced number of concurrent ions in tsDDA mode still have 32% and 25% co-eluting ions that were not triggered for fragmentation in the positive and negative ion mode, respectively. If more metabolites are extracted from the full scan, more ion lists and MS runs will be required.

Overall, based on the reported studies, when only one MS instrument was utilized in hybrid metabolomics analysis, LRMS was generally considered as comparable to or slightly better than HRMS-based metabolomics, as the abundant ions that simultaneously get into the MS detector can often induce signal saturation in HRMS, which in turn could deteriorate the mass accuracy and compromise the advantage of HRMS. Meanwhile, data pre-treatment/processing (before statistical analysis) in LRMS is relatively easy compared to HRMS, and the data size is generally smaller.

#### 2.2.3. The Integration of LRMS and HRMS with Hybrid Approaches

From the reviewed literature, we discovered that researchers can also combine at least two mass spectrometers to develop hybrid approaches for robust, comprehensive analysis. The hybrid usages of two different instruments could increase the number of ion pairs and improve the repeatability for sample analysis in untargeted metabolomics. A diverse set of Q-TOF-MS [37] or Q-Orbitrap-MS [44] with QQQ-MS [31] or QTRAP-MS [40] have been used, and in most cases, HRMS analysis was first operated in the full scan mode. Meanwhile, “auto MS/MS” [37], IDA [31], or the sequential windowed acquisition of all theoretical fragment ions (SWATH) [26] were subsequentially used to acquire the scheduled SRMs. In several example applications [26,39], IDA and SWATH were used to increase the number of characteristic ion pairs, especially at the time of co-elution occurrence or for the detection of low-abundant metabolites. These MS data can also assist the SRM ion-pair selection by providing information regarding the precursor ion, product ion, retention time, and collision energy. Next, the dynamic SRM mode was employed to monitor each ion pair near its expected retention time. Furthermore, systematic and automated software, such as MRM-Ion Pair Finder, can be developed to reduce the time needed to pick ion pairs from thousands of candidates [31]. A comprehensive strategy combining blank-wash, large pooled QC samples, and post-calibration was also developed to improve the stability of the large-scale pseudotargeted metabolomics [28]. Additionally, the same LC conditions and the same UHPLC system across the two types of instruments can be used to avoid the difference of chromatographic separation [40]. In one example study, 76% of the metabolites detected by the pseudotargeted method with two instruments displayed an RSD of less than 10%, while only 44% of the UHPLC-Q-TOF MS-detected metabolites had an RSD of less than 10% in the pooled serum samples. It is also noted that 68% and 44% of the metabolites detected by pseudotargeted metabolomics and untargeted metabolomics, respectively, had a correlation coefficient larger than 0.9 [37]. The results indicated that the hybrid approaches exhibited high repeatability in the original and normalized peak area, which has a clear advantage for time series data. It is also worth noticing that the scheduled SRM mode that was used in this pseudotargeted metabolomics study could effectively reduce the number of interfering ions and enhance the scan rate. Because the detected metabolites have been predefined, no complicated data processing is needed in complex sample analysis. Overall, the hybrid methods discussed in this study demonstrated the utility of combining two instrument platforms from the same vendor for extensive biomarker discovery and quantitative metabolite analysis. In general, the hybrid methods in metabolomics with two instruments could maximize the number of included ion pairs compared to the HRMS or LRMS-only methods, which is considered a major advantage (Table 1). However, compared to the single instrument-based methods, the integration requires two relatively high-end instruments, which makes method transformation inevitable. To ensure the transmissibility of data and ion pairs being identified in both instruments, it is generally a better situation if the two instruments used in the combined approaches come from the same manufacturer. In addition, when HRMS-acquired ion pairs are imported to the LRMS workstation, some instrument parameters (such as fragmentation voltage and collision energy) need to be re-optimized to minimize the changes in the elution order and retention time of the metabolites. However, the usage of two instruments increase the cost of purchasing and maintaining the equipment, and require more lab space for installation. Besides, transforming thousands of candidates between two instruments can be labor-intensive. Therefore, these reasons greatly limit its application.

### 2.3. Data Handling in Hybrid Metabolomics Approches

The post-experiment data-handling in metabolomics studies is usually critical and essential, and there is no exception in novel metabolomics studies with hybrid approaches. Almost all the discussed hybrid studies here manually extracted and aligned the MS peaks through commercial data processing software provided by vendors or online data processing servers/websites, such as Bruker Daltonics Control [59], Bruker Daltonics Data analysis software [60], MarkerLynx XS software [61], Extracted Ion Chromatograms (XICs) [62], XCMS software [63], Thermo Compound Discover and Xcalibar software [64], and Progenesis QI [65]. A homemade software [31] was used in the hybrid study, which developed an automated and reliable approach, MRM-Ion Pair Finder, to accelerate this key process and to improve the data quality of the global metabolome measurements. Besides this, the programming language in which a software is written can increase the flexibility in creating a customized workflow and capacity in dealing with large data, such as the R package [66,67] and Phyton package [68]. Before multivariate and univariate analyses, the data were also commonly pretreated to be suitable for analysis [69,70]. These methods include the log10 transformation; the internal standard-based normalization; and the annotation by databases, such as METLIN, HMDB, BiGG, SetupX, BinBase, and the MetaboLights database (http://metabolomicssociety.org/resources/metabolomics-databases). Metabolite data from the hybrid analyses could also be filtered in terms of their dynamic range, linearity, reproducibility, coverage, and metabolic feature distribution with author-preferred thresholds. Meanwhile, the evolution in MS technologies has constantly increased the complexity and the size of MS data that can be generated from many biological analyses. Then, both univariate (e.g., student’s t-test) and multivariate (e.g., principal component analysis and partial least squares discriminant analysis [71]) statistical analyses have been applied to comprehensively analyze the data from hybrid analyses [72]. Statistical software and platforms such as SPSS [40,73], GraphPad Prism [27,74], SIMCA-P [36,75], and MetaboAnalyst (https://www.metaboanalyst.ca/) [43] have also been used to assist the data analysis and result interpretation. In summary, many data analysis techniques, software, and platforms have been used in hybrid approaches.

## 3. Representative Applications of Hybrid Approaches in Metabolomics

Over the past few years, hybrid approaches in metabolomics have been applied in biological samples that are associated with different kinds of disease, such as cardiovascular disease [76], neurodegenerative disease [77], cancer [39,40,41], kidney dysfunction [78], and diabetes mellitus [55]. The established hybrid methods discussed above also have been used in a variety of biological samples (such as urine, serum/plasma, and tissue samples) to facilitate broader metabolite coverage or to validate metabolite biomarkers for disease. For example, a study performed by Liu and colleagues used a novel strategy combining isotope labeling with LC-MS in the double precursor ion scan and MRM/SRM mode for the untargeted profiling and targeted quantitation of thiols from urine samples of patients with five types of cancer (nasopharyngeal cancer, esophagus cancer, gastric cancer, lymph cancer, and lung cancer). One hundred and three thiol candidates were discovered in all the cancers, and six thiols were identified by chemical standards, of which pantetheine was identified in human urine for the first time. Additionally, the concentrations of homocysteine, γ-glutamylcysteine, and pantetheine were detected with more than two-fold increases in cancer patients compared to healthy controls [41].

Meanwhile, the hybrid approaches in metabolomics was also used in biomarker discovery for hepatocellular carcinoma (HCC) and bladder cancer diagnosis [39]. To identify potential metabolite biomarkers for HCC diagnosis, a urinary pseudotargeted method based on LC−QTRAP-MS was developed, and the authors found that urinary nucleosides, bile acids, citric acid, and several amino acids were significantly changed in liver disease groups compared with the controls, featuring the dysregulation of purine metabolism, energy metabolism, and amino metabolism in liver diseases. Similarly, butyrylcarnitine (carnitine C4:0) and hydantoin-5-propionic acid were defined as combinational markers to distinguish HCC from cirrhosis with the pseudotargeted method [40]. Furthermore, Chen and colleagues identified 50 serum metabolite biomarkers from their HCC samples with the pseudo-targeted approach, which demonstrated that patients with HCC had lower amounts of lysophosphatidylcholines, higher amounts of long chains, decreased amounts of medium-chain acylcarnitines, higher amounts of aromatic amino acids, and lower branched-chain amino acids levels than healthy controls [37]. Interestingly, the discovered and validated serum metabolite biomarker panel (including phenylalanyl-tryptophan and glycocholate) reported by Luo and colleagues also exhibited a good diagnostic performance for the early detection of HCC from at-risk populations [54]. When it comes to bladder cancer (BC) study, a urinary pseudotargeted method based on GC-MS was developed and validated, and a total of 76 differential metabolites were detected in the discovery sample set, 58 of which were verified using an independent validation set. Based on their further analysis, a four-biomarker panel was defined for the general diagnosis of BC. Besides this, the combinatorial biomarker panel also proved useful for the early diagnosis of BC. This study proved that the proposed pseudo-targeted approach can be used to discriminate non-muscle invasive and low-grade BCs from healthy controls with a satisfactory sensitivity and specificity, and that this method can be employed to effectively and relatively noninvasively screen BC biomarkers [39]. In addition to cancer research, a pseudotargeted method based on UHPLC−MS with SWATH acquisition was also applied to a Type 2 diabetes study. In this study [55], 162 significantly changed metabolites were defined in the serum of type 2 diabetes patients, which demonstrated that the pseudotargeted method can provide many useful hints for investigating changes in metabolites and has great potential in promoting our understanding of the metabolism processes of metabolic disease.

Beyond human disease investigations, the GOT-MS approach was also used in bacterial metabolism analysis. The human gut microbiota plays an important role in human physiological processes such as nutrient digestion and the regulation of the immune system. Thus, examining the metabolites from the gut microbiota can provide a better understanding of the activity of gut microbes, and further inform us of their impact on human health. A recent study demonstrated the application of an innovative secondary electrospray ionization (SESI)-GOT-MS/MS method in the investigation of gut microbial metabolism in vitro [29], while 71 features in the SESI-GOT-MS/MS method are potentially new metabolites that exist in the headspace of gut microbial culture. In another bacterial analysis, the metabolic profiling-based differentiation of methicillin-susceptible and methicillin-resistant *S. aureus* bacteria was achieved by ts/msGOT-MS with a better analytical performance than targeted metabolomics, as the detected differential metabolites by the targeted metabolomics profiling, tsGOT-MS, msGOT-MS were reported as 50, 214, and 260, respectively [45].

Plant metabolomics has been widely applied to plant physiological metabolism [79], abiotic and biotic stress responses [80], etc. The pseudo-targeted methods can also be used for the metabolic profiling analysis of plants. For instance, the pseudotargeted UHPLC-QQQ-MS dynamic MRM method was used for the investigation of metabolic variations in rice seeds with two wild cultivars [28]. A total of 749 and 617 ion pairs in positive and negative modes were achieved, respectively. Among them, about 200 metabolites were identified or tentatively identified. Another pseudotargeted method using GC-MS-selected ion monitoring was applied to investigate the chemical characteristics of commercial cigarettes made in China and foreign countries [36]. In this study, a peak table with 312 components and their related quantitation ions was generated for SIM acquisition, and a total of 90 compounds were identified. Their results indicated that Chinese domestic flue-cured cigarettes have a higher concentration of saccharides and a lower concentration of organic acids and amino acids than domestic blended cigarettes and foreign cigarettes.

## 4. Conclusions and Future Perspectives

**Figure 3:**
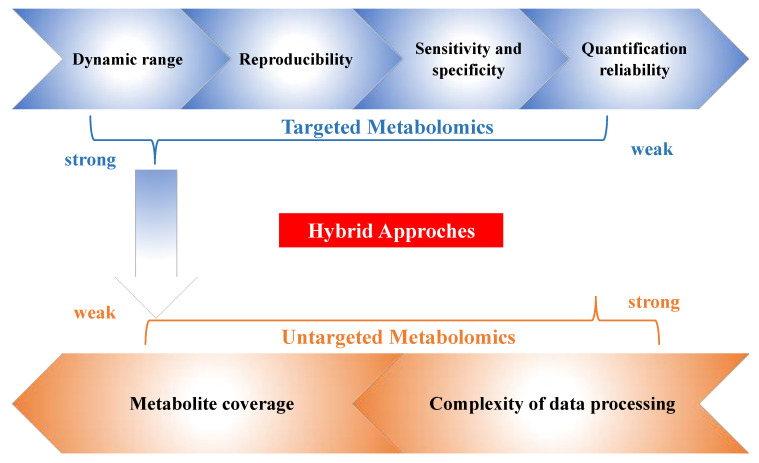
Considerations for novel metabolomics analysis with hybrid approaches with advantages from both untargeted and targeted analysis.

From the existing body of literature, we have demonstrated that the hybrid approach provides us a new avenue to expand the metabolome coverage in parallel with targeted analysis. It maintained some strengths of targeted and untargeted metabolomics analysis, while neutralizing some of their weaknesses (Figure 3). It can be used to distinguish groups of samples with different metabolic features; it has great potential in biomarker discovery, disease diagnosis, and quantitative work using mixed linear calibration. While promising, the major limitation of the hybrid metabolomics methods remains. For example, the relatively longer sample running times can be a bottleneck for truly high-throughput analysis, especially when dealing with valuable samples that have limited availability. Future experiments using a faster and more sensitive instrument will potentially decrease the run time and increase the analysis throughput. Furthermore, the extensive need for instrument method optimization and careful result de-duplication can also slow down the hybrid analysis process. Thus, to generate a “one feature for one peak” dataset, the effective and comprehensive instrument method setup and data filtering procedure remain to be exploited. Besides, a seamless integration of in-house hybrid approaches results in publicly available databases are not always available, which posited challenges in confident and accurate compound identification. The possible solutions for this current weakness include increasing the number of metabolites in the in-house database or improving the integration of the novel metabolomics data analysis workflow with available online databases. Hopefully, with the prevalence of hybrid metabolomics studies, more datasets with detailed fragmentation information will become available, which will facilitate further annotation efforts for many unknowns.

## Figures and Tables

**Table 1:** Summary of the performance and analytical considerations from different hybrid approaches.

Instrumentation	Biological Sample	Method Validation	Metabolites Coverage and RT (min)	Data Processing Strategies	Ref
UHPLC-Q-TOF-MS and UHPLC-QTRAP-MS	Human urine	69% and 94% of metabolites displayed a relative standard deviation (RSD) of <10% and <20%, respectively. After normalization and internal standard calibration, 94% and 97% of metabolites had an RSD of < 20%, respectively.	419 compounds in electrospray ionization quadrupole (ESI) positive mode and 449 compounds in ESI negative mode were detected. RT = 26 min.	Analyst 1.6 software (AB SCIEX, USA) was used to conduct peak integration. The non-targeted analysis data were imported to the SIEVE software (ThermoFisher, USA) package to extract the metabolite features.	[40]
LC-QTRAP-MS	Human urine	The slopes of linear regressions were approximately 1.00 (1.0291). RSDs were less than 9.0% with a correlation coefficient (R^2^) of 0.9998.	The number of accurately quantified metabolites from 103 thiols, increased from 64 (62%) to 99 (96%). RT = 55 min.	Manual data acquisition and processing through Bruker Daltonics Control 3.4 and Bruker Daltonics Data analysis 4.0 software (Bruker Daltonics, Bremen, Germany).	[41]
GC-MS	Human urine	75.7%, 89.5%, and 95.9% of the peaks had RSDs of <10%, 20%, and 30%, respectively. The intraday RSDs for 80.2%, 91.6%, and 95.1% of the peaks were <10%, 20%, and 30%, respectively. The interday RSD for 64.3%, 86.9%, and 91.6% of the peaks were <10%, 20%, and 30%, respectively.	A total of 76 differential metabolites were defined, 58 of which were verified. RT = 30 min.	Changes in the levels of the differential metabolites were visualized with MultiExperiment Viewer (http://www.tm4.org). The altered pathways were determined with MetaboAnalyst 2.0 (http://www.metaboanalyst.ca).	[52]
UHPLC-Q-Orbitrap-MS and UHPLC-QTRAP-MS	Human urine	The RSDs of all the standards were below 10%. 94% and 80% of the peaks had RSDs of <30% and <20% in the QC samples, respectively. The 95.5% and 90% peaks had RSDs of <30% and <20% in the urine samples, respectively.	780 metabolites were defined. A total of 48 metabolites were chosen, 26 of which were identified. RT = 28 min.	Ion-pairs selection was from two sources: (1) untargeted metabolic analysis; (2) key metabolites in the metabolic pathways chosen from free databases. MultiQuant software (Applied Biosystem/MDS Sciex, Carlsbad, CA) was used to extract the peaks.	[53]
UHPLC-Q-TOF-MS and UHPLC-QQQ-MS	Human serum	34% of the detected metabolites had an RSD < 5% and 76% had an RSD < 10%.	518 ion-pairs were defined for subsequent MRM detection. RT = 29.9 min.	Manual peak detection and alignment through XCMS software.	[37]
LC-QTOF-MS	Human serum	The minimum absolute height required was set at 3000 counts, which was also used for 100% of analyses in the recursive step to minimize the experimental variability.	139 and 158 molecular entities in the negative and positive ionization mode, respectively, were obtained. RT = 20 min.	The MassHunter Workstation software package was used to process all the data obtained by LC/Q-TOF in the MS/MS mode.	[38]
UHPLC-Q-TOF-MS and UHPLC-QQQ-MS	Human serum	91% of contents had an RSD of <30% from the QC samples.	1446 metabolite MRM transitions in the MRM-Ion Pair Finder. RT = 23 min.	Efficient data processing strategy, with the processing time markedly shortened by the homemade MRM-Ion Pair Finder software.	[31]
LC−QQQ-MS	Human serum	The linearity r at 0.82 ± 0.26 of amino acids. >40% of the GOT-MS MRMs had CVs < 5%; the average CV of the detected metabolites was at 7.8 ± 7.0%.	595 precursor ions and 1890 multiple reaction monitoring transitions (MRM). RT = 9 min.	Manual inspection based on symmetry, peak width, and MS peak area extraction by Agilent MassHunter Qualitative Analysis and Quantitative Analysis software (Agilent Technologies, Inc., Santa Clara, CA).	[24]
UHPLC-Q-TOF-MS and UHPLC/QTRAP-MS	Human serum	The change folds of a peak area between these two QC samples ranged from 0.002 to 14. 767 (94.3%) and 759 (93.3%) of the peaks had a CV of <30% in the b-QC and p-QC replicates, respectively.	813 ions were steadily detected in the QC samples. RT = 30 min.	The integrations of the peak areas were processed by the software provided by the instrument vendor. The efficient data processing strategy used the MRM-Ion Pair Finder software.	[28]
UHPLC-QTRAP-MS and LC-MS	Human serum	For the validation set, the AUC was from 0.676 to 0.875. Sensitivity was from 0.504 to 0.921. Specificity was from 0.528 to 0.784.	239 metabolites were identified. RT = 20 min.	Manual peak detection and alignment through the XCMS software. Home-developed database and online databases (HMDB and Metlin) or confirming with authentic standards were used.	[54]
2 D-UHPLC-QTRAP-MS	Human plasma	The linear regression r varied between 0.9902 and 0.9993. The average accuracies for the standard samples were between 0.11% and 13.90%, and the average intra-/inter-day precisions of the standard samples were between 0.66% and 16.46%. The intra-/inter-day precisions in the complex plasma matrix were between 2.05% and 19.49%.	78 metabolites were confidently confirmed, from which 73 metabolites can be accurately quantified. Untargeted profiling of 4651 features of high reliability and validity was achieved. RT = 27 min.	Targeted qualitative and quantitative was performed using the TraceFinder 3.3 software (Thermo Fisher Scientific, Waltham, MA). All the calibration curves were linear and weighted 1/x. The untargeted analysis was performed by the Progenesis QI 2.0 software (Waters, Milford, MA, USA).	[26]
UHPLC-Q-Orbitrap-MS and UHPLC-Qtrap-MS	Human plasma	43 yielded good linear functions (R2 > 0.99) had RSDs lower than 20% and an accuracy between 80% and 120%.	1658 characteristic ion-pairs from 1324 metabolites. RT = 35 min.	48 metabolites established DITQM; manually generate “one feature for one peak” metabolomics data, further confirmed through the extracted ion chromatograms (XICs).	[39]
UHPLC-Q-TOF-MS and UHPLC-QTRAP-MS	Human plasma	The intraday RSDs of 82.4% of the metabolites were <15%. 25.1% of the metabolites had RSD values of >15%.	A total of 1373 unique metabolite ion-pairs were obtained in the positive ion mode. 162 significantly changed metabolites were defined. RT = 30 min.	Peak detection and alignment were performed by the MarkerView software (AB SCIEX, Framingham, USA) MS-DIAL software and homemade C-package were used to handle MS2. UHPLC-MRM MS data were disposed of in Analyst 1.6 software (AB SCIEX, Framingham, USA).	[55]
UHPLC-QQQ-MS	Human plasma	The optimized MRMs had intensities of >1000 and signal-to-noise-ratios (S/Ns) of >3. The intraday and interday median CVs were 4.86% and 8.79%, respectively. The median r was 0.96.	927 metabolites were measured. 310 were confirmed using pure chemical standards, while the rest were annotated by identification level using database entries. RT = 15 min.	The entire LC-MS system was controlled by the Mass Hunter Workstation software (Agilent, Santa Clara, CA). The extracted MRM peaks were integrated using the Mass Hunter Quantitative Data Analysis software.	[51]
UHPLC-Q-TOF-MS	Mouse serum	66.7% of metabolites displayed an RSD of <10%. 99.8% of metabolites had an RSD of <20%. After normalization by the total intensity and sample median, 99.8% and 99.4% of metabolites exhibited an RSD of <20%, respectively	2081 ions were obtained after data filtering. 569 peaks were selected to perform tsMIM-based pseudotargeted analysis. RT = 26 min.	The raw data were extracted and aligned by the Progenesis QI software. The peak areas were collected by the TargetLynx software.	[43]
UHPLC-Q-TOF-MS	Mouse plasma	97.4% and 95.4% of the selected ions in the positive and negative ion modes have RSD values of less than 20%, respectively.	1423 and 1141 ions were generated in the positive and negative ion modes, respectively. RT = 27 min.	The raw data files were uploaded into the Progenesis QI software to perform chromatographic peak alignment, data normalization, and peak picking using the default settings. MassLynx XS (Waters Corp., Manchester, UK) was used for the targeted extraction. The selected features were tentatively annotated.	[42]
UHPLCQ-TOF-MS	Mouse plasma	Almost 90% of the tsMIM- detected metabolites had an RSD of <20%. 42% of metabolites had RDSs of <5%.	387 ions were detected. 17 metabolites were selected as biomarkers. RT = 26 min.	The peak areas of the metabolites were processed using the TargetLynx software.	[27]
UHPLC-Q-Orbitrap-MS and UHPLC-QQQ MS	Mouse brain and liver, cancer cells, and human plasma	The linear of IS r > 0.99. The intraday RSDs were from 0.8% to 4.3%; the inter-day RSDs were from 2.4% to 16.8%. 51% and 94% of the detected lipids had RSDs of <5% and 20% in positive mode and 94% had RSDs of <10% in negative mode.	A total of 3377 targeted lipid ion pairs with over 7000 lipid molecular structures were defined. RT = 20 min.	The raw data were processed with Analyst software. Normalization of the lipids by the appropriate lipid ISs.	[44]
UHPLC-Q-TOF-MS and UHPLC-QTRAP-MS	Rice seed	89.5% of peaks had RSDs < 20%. The linear range was 2.5–4 orders of magnitude, and the r was in the range 0.996–0.999. The recoveries were 85.9–106.3% for positive mode and 73.3–98.2% for negative mode, respectively.	A total of 749 and 617 ion pairs in the positive and negative modes were achieved, respectively. Among them, about 200 metabolites were identified or tentatively identified. RT = 30 min.	All the ions were extracted by the Analyst software. Zero values were removed by the 80% rule.	[28]
GC-MS	Commercial cigarettes	>81.2% of peaks had RSDs of <20%.	312 components and their related quantitation ions. A total of 90 compounds were elucidated. RT = 72.5 min.	The integration of the chromatography peaks was performed using the Agilent MSD ChemStation (Agilent Technologies). Peak areas of all the components were divided by those of the internal standard and then scaled to zero mean and unit variance.	[36]
GC-MS	Tobacco leaf	47.3% of components had an r of >0.99; 36% of components had an RSD of <5%; 93% of components had an RSD of < 20%.	167 differential components (*p* < 0.05) were screened out. RT = 72.5 min.	The acquired GC/MS raw data of the QC sample were imported into the AMDIS software (version 2.62, NIST, USA) for peak deconvolution and detection. The quantitative ion selection algorithm was written in Visual C++ (version 6.0, Microsoft). The component peak area was divided by that of the internal standard.	[25]
SESI-QQQ-MS	Bacterial culture	47% of features showed an r of >0.90. 65.9% of features had a CV of <20%	75 features in the SESI-GOT-MS/MS panel were established. RT = 1 min.	Manual inspection based on symmetry, peak width, and MS peak area extraction by Agilent MassHunter Qualitative Analysis and Quantitative Analysis software.	[29]
UHPLC-QQQ-MS	Bacterial culture	54.9% of peaks had measurement CVs of <10%; 0.84% had CVs of >40%. 51.97% of peaks had CVs of <10%; 0% had CVs of >40%.	A total of 464 metabolite peaks were detected. RT = 20 min.	All the raw metabolomics data were inspected by the Quanbrowser module of Xcalibur version 4.0 (Thermo Fisher Scientific) and the Thermo TSQ LC-SIM Data Processor (V0.1.35 Engineering Sample).	[45]

## Abbreviations

AMDIS	automated mass spectral deconvolution and identification system
BC	bladder cancer
cv	coefficient of variation
DDA	data-dependent acquisition
tDDA	target-direct data-dependent acquisition
tsDDA	time staggered-direct data-dependent acquisition
DITQM	data independent targeted quantitative metabolomics
EIC	extracted ions current
ESI	electrospray ionization quadrupole
GC	gas chromatography
GC-MS	gas chromatography coupled with mass spectrometry
GOT	globally optimized targeted method
dGOT	database-assisted globally optimized targeted method
ts/msGOT	time staggered/mass staggered globally optimized targeted method
GPF	gas-phase fractionation method
HCC	hepatocellular carcinoma
HMDB	human metabolome database
HPLC	high-performance liquid chromatography
UHPLC	Ulrta-high performance liquid chromatography
HPLC-MS	high-performance liquid chromatography coupled with mass spectrometry
HRMS	high resolution mass spectrometers
IDA	information dependent acquisition
LRMS	low resolution mass spectrometers
MIM	multiple ion monitoring
tsMIM	time staggered-multiple ion monitoring
MRM	multiple reaction monitoring
MS	mass spectrometry
NMR	nuclear magnetic resonance
Q-Orbitrap	quadrupole-orbitrap
Q-TOF	quadrupole time-of-flight
QC	quality control
QQQ MS	triple quadrupole mass spectrometry
QTrap	quadrupole trap
RSD	Relative Standard Deviation
RT	retention time
RTL	the retention time locking
SESI	secondary electrospray ionization
SIM	selected ion monitoring
SRM	selected reaction monitoring
SWATH	sequential windowed acquisition of all theoretical fragment ion
TIC	total ion current
TOF	time of flight
